# Loss of muscle strength in community-dwelling elderly is associated with type 2 diabetes

**DOI:** 10.1186/1758-5996-7-S1-A39

**Published:** 2015-11-11

**Authors:** Valéria Pagotto, Daniella Moreira Dias, Kassyla Ferreira dos Santos, Joyce Gabriella Menezes Silva, Wanessa Freitas Silva, Erika Aparecida da Silveira

**Affiliations:** 1 Universidade Federal de Goiás, Goiânia, Brazil

## Background

A loss of skeletal muscle mass and strength is frequently observed in older adults. Persons with diabetes have accelerated muscle and strength loss, but the relationship of hyperglycemia to declines in muscle function has not been explored yet.

## Objective

To investigate the relationship between type 2 diabetes and low muscle mass and strength in community-dwelling elderly.

## Materials and methods

Data was obtained in the Elderly Project/Goiânia, a cross-sectional study comprising 132 community dwelling elderly (>60 yrs.) of Goiânia, Goiás, Brazil. Muscle mass was estimated by standard methods by using dual-x-ray-absortiometry (DXA) and was determined by the skeletal muscle mass index (SMI). Muscle strength was determined by Hand grip strength (hand-GS) with a dynamometer on dominant hand. Diabetes diagnose was identified by self-report or use of hypoglycemic agents. Analyses were performed in STATA 12.0. We calculated the prevalence and the difference ratio was evaluated by the Pearson chi-square test (p <0.05). This study was approved by the Research Ethics Committee of UFG.

## Results

Of the 132 elderly studied, 60.9% were women, mean age 70.1 yrs. (±6.63) and mean BMI of 26.7 kg/m2 (±26.7). The mean SMI was 6.69 kg/m^2^, and 7.50 kg/m^2^ among men and 6.16 kg/m^2^ in women (p=0.001). The average FPP was 22.8 kgf (±8.38), and 29,9kgf between men and 18,1kgf in women (p=0.001). The prevalence of diabetes was 18.5%, with higher prevalence in young people aged, 60-69 yrs. (21.7%). Low muscle strength was observed in 25% of diabetic patients (p=0.05) and low muscle mass in 8.3% of diabetics with no statistically significant differences (p>0.05).

## Conclusions

Type 2 diabetes is associated with loss of skeletal muscle strength in community-dwelling older adults. Future studies should explore if better glycemic control can preserve muscle strength in diabetes.

